# Effectiveness and safety of oral anticoagulants in non-valvular atrial fibrillation patients with prior bleeding events: a retrospective analysis of administrative claims databases

**DOI:** 10.1007/s11239-022-02660-2

**Published:** 2022-05-17

**Authors:** Gregory Y. H. Lip, Allison Keshishian, Amiee Kang, Xuemei Luo, Nipun Atreja, Yan Zhang, Patricia Schuler, Jenny Jiang, Huseyin Yuce, Steven Deitelzweig

**Affiliations:** 1 Liverpool Centre for Cardiovascular Science, University of Liverpool and Liverpool Heart & Chest Hospital, William Henry Duncan Building 6 West Derby Street, L7 8TX Liverpool, United Kingdom; 2 Aalborg Thrombosis Research Unit, Department of Clinical Medicine, Aalborg University, Aalborg, Denmark; 3 STATinMED Research, Ann Arbor, MI USA; 4 New York City College of Technology, City University of New York, New York, NY United States; 5 Bristol-Myers Squibb Company, Lawrenceville, NJ USA; 6 Pfizer, Inc, Groton, CT USA; 7 Department of Hospital Medicine, Ochsner Clinic Foundation, New Orleans, LA USA; 8 The University of Queensland School of Medicine, Ochsner Clinical School, New Orleans, LA United States

**Keywords:** Major bleed, Nonvalvular atrial fibrillation, Non-vitamin K anticoagulant, Warfarin

## Abstract

**Introduction:**

There are a paucity of real-world data examining effectiveness and safety of non-vitamin K antagonist oral anticoagulants (NOACs) and warfarin in nonvalvular atrial fibrillation (NVAF) patients with prior bleeding.

**Methods:**

This retrospective analysis included data from 5 insurance claims databases and included NVAF patients prescribed OACs with prior bleeding. One-to-one propensity score matching was conducted between NOACs and warfarin and between NOACs in each database. Cox proportional hazards models were used to evaluate the risk of stroke/systemic embolism (SE) and MB.

**Results:**

A total of 244,563 patients (mean age 77; 50% female) with prior bleeding included 55,094 (22.5%) treated with apixaban, 12,500 (5.1%) with dabigatran, 38,246 (15.6%) with rivaroxaban, and 138,723 (56.7%) with warfarin. Apixaban (hazard ratio [HR]: 0.76 [95% CI: 0.70, 0.83]) and rivaroxaban (HR: 0.79 [95% CI: 0.71, 0.87]) had a lower risk of stroke/SE vs. warfarin. Apixaban (HR: 0.67 [95% CI: 0.64, 0.70]) and dabigatran (HR: 0.88 [95% CI: 0.81, 0.96]) had a lower risk of MB vs. warfarin. Apixaban patients had a lower risk of stroke/SE vs. dabigatran (HR: 0.70 [95% CI: 0.57, 0.86]) and rivaroxaban (HR: 0.85 [95% CI: 0.76, 0.96]) and a lower risk of MB than dabigatran (HR: 0.73 [95% CI: 0.67, 0.81]) and rivaroxaban (HR: 0.64 [95% CI: 0.61, 0.68]).

**Conclusions:**

In this real-world analysis of a large sample of NVAF patients with prior bleeding, NOACs were associated with similar or lower risk of stroke/SE and MB vs. warfarin and variable risk of stroke/SE and MB against each other.

**Supplementary information:**

The online version contains supplementary material available at 10.1007/s11239-022-02660-2.

## Highlights

- Data on NOAC effectiveness and safety in NVAF patients with prior bleed history are lacking.
- This study included data on OAC-treated NVAF patients with a history of bleeding.
- NOACs were associated with similar or lower risk of stroke/SE and MB vs. warfarin.
- NOACs were associated with variable risk of stroke/SE and MB against each other.
- This study further demonstrated the effectiveness and safety profile when comparing NOACs to warfarin. The findings could aid to inform the discussion on the benefits and risks in the decision making process for NVAF patients who had a prior bleed.

**Supplementary information:**

The online version contains supplementary material available at 10.1007/s11239-022-02660-2.

## Introduction

Atrial fibrillation (AF) is the most commonly treated cardiac arrhythmia globally, with a major impact on healthcare costs.[1] The high risk of stroke and mortality following AF diagnosis is concerning. In emergency department settings, about 4% of patients experience stroke within one year of AF diagnosis, and about 11% die within that same time frame (8% due to stroke).[2] The complexity of AF needs a holistic approach with multidisciplinary, integrated management with active involvement of AF patients.[3] This integrated approach to patient evaluation and management is increasingly advocated for AF patients[4] given the beneficial impact on clinical outcomes.[5, 6].

A history of bleeding in the context of AF presents challenges for clinical management. AF patients with prior serious hemorrhagic events, like gastrointestinal (GI) bleed or intracranial hemorrhage (ICH), are at an increased risk for subsequent serious hemorrhagic events.[7, 8] Resumption of anticoagulation therapy in AF patients following a major bleeding (MB) event may lower the risk of ischemic events and all-cause mortality[7, 9–14]; however, studies have found a high risk of recurrent MB when resuming oral anticoagulants (OAC), [13–16] thus presenting a clinical challenge. The clinician must therefore weigh the antithrombotic benefits of anticoagulation therapy against the possibility of incurring another major hemorrhagic event should therapy resume.

Vitamin K antagonists (VKAs), such as warfarin used to be the standard of care for stroke prevention in patients with non-valvular AF (NVAF).[17] The advent of the non-vitamin K antagonist OACs (NOACs) apixaban, dabigatran, edoxaban, and rivaroxaban has provided a convenient, efficacious, and tolerable alternative to anticoagulation with warfarin.[18] Unsurprisingly, the NOACs are increasingly used in everyday clinical practice.[19, 20].

Because of these differences, it is essential to evaluate whether AF patients with a history of bleeding might have different outcomes when they are treated with NOACs vs. warfarin. Additionally, as the uptake of NOACs continues to increase, more data will be needed to fully understand the risk–benefit profiles associated with each NOAC.

To date, research about anticoagulant therapy in AF following a major hemorrhagic event has generally focused on warfarin therapy alone or warfarin vs. NOACs collectively rather than comparing the individual NOACs to warfarin or to one another.[7, 12, 21–23] This is a critical omission, as pharmacokinetic differences among NOACs may affect their respective efficacy and safety. Further, given that the effectiveness and tolerability of pharmacotherapy in patients with NVAF can be influenced by pre-existing patient comorbidities, such as a history of bleeding, information on this specific subset of the AF population could be significant when making therapeutic decisions. To help address these gaps, this study assessed stroke/SE and MB associated with NOACs vs. warfarin and vs. one another among NVAF patients with prior bleeding.

## Methods

### Data sources

This was a retrospective observational data analysis of NVAF patients with a history of bleeding who received treatment with NOACs (i.e., apixaban, dabigatran, edoxaban, or rivaroxaban) or warfarin. Data were pooled from a sample of more than 180 million beneficiaries (~ 56% of the US population) using the five largest insurance databases in the US: Fee-for-Service Medicare data from the U.S. Centers for Medicare & Medicaid Services (CMS), the IBM Watson Health MarketScan Commercial Claims and Encounter (“MarketScan”), the IQVIA PharMetrics Plus™ Database (“PharMetrics”), the Optum Clinformatics™ Data Mart (“Optum”), and the Humana Research Database (“Humana”). Patients with Medicare Fee-for-Service, Medicare Advantage, and commercial insurance were included. Database records included demographic and clinical information and *International Classification of Diseases, Ninth and Tenth Revision, Clinical Modification* (ICD-9-CM and ICD-10-CM) codes, Healthcare Common Procedure Coding System (HCPCS) codes, and National Drug Codes.[24].

### Patient selection

**Fig. 1:**
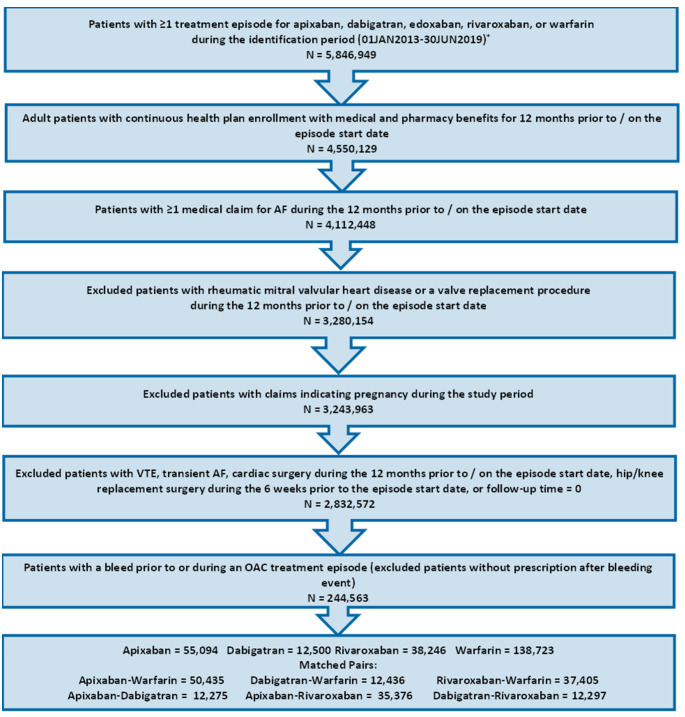
Patient Selection Criteria

Adult patients (age ≥ 18 on index date) with an OAC treatment episode (apixaban, dabigatran, edoxaban, rivaroxaban, or warfarin) between January 1, 2013 and June 30, 2019 (identification period) were selected. A treatment episode was defined as the treatment from OAC prescription date to discontinuation (> 30 days with no OAC use), switch, death, the end of study period, or end of continuous medical or pharmacy enrollment. Episodes were included if the patient had an AF diagnosis during the 12 months prior to / on the OAC prescription date and continuous medical and pharmacy health plan enrollment for 12 months before or on the OAC prescription date (baseline period). Episodes were excluded if the patients had evidence of valvular heart disease, venous thromboembolism, transient AF (pericarditis, hyperthyroidism, thyrotoxicity), or heart valve replacement during the baseline period; were pregnant during the study period; or underwent hip or knee replacement surgery within 6 weeks before the OAC prescription date. Additional patient selection criteria are provided in Fig. 1.

Among patients with eligible OAC episodes, those with a bleeding event prior to or during the OAC treatment episode were selected. A *bleeding event* was defined as a hospitalization with a diagnosis of ICH or GI bleeding or a hospitalization with a primary diagnosis of bleeding at other key sites (e.g., conjunctival, genitourinary system, hematuria. The full list of codes used for other key sites can be found in Supplemental Table 1). If a patient had more than one type of bleed during baseline, a hierarchy was applied to categorize patients as follows: ICH, GI, and bleeding at other key sites. The first OAC prescription date after a bleeding event was designated as the index date. If bleeding event occurs during an OAC episode, the index date reflects the first prescription after the bleeding event within a treatment episode. If the bleeding event occurs prior to OAC treatment episode, the index date reflects the start of an OAC treatment episode. Patients prescribed edoxaban were excluded due to small sample size.

### Outcome measures

The primary effectiveness outcome was stroke/systemic embolism (SE), stratified by stroke type (i.e., ischemic, hemorrhagic, and SE). The primary safety outcome was MB, stratified by GI bleeding, ICH, and MB in other key sites.[25, 26] Primary outcomes were operationalized by inpatient claims with stroke/SE or MB as the principal (Medicare, MarketScan, and Optum) or first-listed (Humana and PharMetrics) diagnosis. Diagnosis codes for stroke/SE and MB are presented in Supplemental Table 1.

Outcomes were assessed during the follow-up period, defined as the time from 1 day post-index date to the earliest of the following: 30 days post-discontinuation date, switch date (date of new OAC within 30-days of end of days supply of index OAC), date of death (inpatient and all-cause death for commercial data and Medicare populations, respectively), end of continuous health plan enrollment, or study end (June 30, 2019).

### Statistical analysis

Descriptive analysis was conducted for each treatment cohort. To control for different patient characteristics, propensity score matching (PSM) was used to compare each individual NOAC with warfarin (i.e., apixaban vs. warfarin, dabigatran vs. warfarin, and rivaroxaban vs. warfarin) as well as each individual NOAC with one another (i.e., apixaban vs. dabigatran, apixaban vs. rivaroxaban, and dabigatran vs. rivaroxaban). PSM was conducted in each database using two comparative cohorts before pooling the datasets. Patients were matched 1:1 by propensity scores generated using multivariable logistic regressions for baseline characteristics, including type of prior bleed, prior OAC use, demographics, and clinical characteristics (see Tables 1 and 2 for complete covariate list). Further details on PSM methodology appear in prior publications.[27] The PSM-adjusted baseline variables were compared based on standardized differences, with a threshold of 10%.[28].

**Table 1:** Baseline Characteristics of Patients Prescribed NOACs vs. Warfarin after Propensity Score Matching

	Apixaban Cohort (N = 50,435)		Warfarin Cohort (N = 50,435)			Dabigatran Cohort (N = 12,436)		Warfarin Cohort (N = 12,436)			Rivaroxaban Cohort (N = 37,405)		Warfarin Cohort (N = 37,405)	
	**N/** **Mean**	**%/** **SD**	**N/** **Mean**	**%/** **SD**		**N/** **Mean**	**%/** **SD**	**N/** **Mean**	**%/** **SD**		**N/** **Mean**	**%/** **SD**	**N/** **Mean**	**%/** **SD**
Age*	77.77	9.06	77.69	9.18		77.56	8.55	77.40	8.76		77.22	9.03	77.28	9.03
**18–54**	507	1.01%	548	1.09%		109	0.88%	138	1.11%		480	1.28%	423	1.13%
**55–64**	1,931	3.83%	1,898	3.76%		434	3.49%	429	3.45%		1,550	4.14%	1,469	3.93%
**65–74**	15,427	30.59%	15,644	31.02%		3,914	31.47%	3,996	32.13%		11,962	31.98%	12,216	32.66%
**75–79**	11,136	22.08%	11,094	22.00%		2,847	22.89%	2,765	22.23%		8,408	22.48%	8,333	22.28%
**≥ 80**	21,434	42.50%	21,251	42.14%		5,132	41.27%	5,108	41.07%		15,005	40.11%	14,964	40.01%
Gender*														
**Male**	24,429	48.44%	24,464	48.51%		6,261	50.35%	6,334	50.93%		18,204	48.67%	18,211	48.69%
**Female**	26,006	51.56%	25,971	51.49%		6,175	49.65%	6,102	49.07%		19,201	51.33%	19,194	51.31%
**Baseline Comorbidity**														
Deyo-Charlson Comorbidity Index*	5.27	3.30	5.28	3.39		4.81	3.14	4.84	3.22		4.93	3.22	4.94	3.30
**CHA**_**2**_ **DS**_**2**_ **-VASc Score**	5.32	1.76	5.40	1.78		5.32	1.77	5.29	1.75		5.24	1.79	5.28	1.77
**0**	64	0.13%	55	0.11%		16	0.13%	15	0.12%		84	0.22%	59	0.16%
**1**	552	1.09%	577	1.14%		145	1.17%	151	1.21%		493	1.32%	483	1.29%
**2**	2,163	4.29%	1,998	3.96%		540	4.34%	488	3.92%		1,780	4.76%	1,589	4.25%
**3**	4,984	9.88%	4,835	9.59%		1,250	10.05%	1,285	10.33%		4,089	10.93%	3,915	10.47%
**4+**	42,672	84.61%	42,970	85.20%		10,485	84.31%	10,497	84.41%		30,959	82.77%	31,359	83.84%
**HAS-BLED Score** ^†^	4.19	1.21	4.22	1.24		4.15	1.16	4.14	1.15		4.12	1.20	4.13	1.21
**0**	37	0.07%	43	0.09%		7	0.06%	7	0.06%		40	0.11%	43	0.11%
**1**	1,107	2.19%	1,296	2.57%		232	1.87%	223	1.79%		851	2.28%	934	2.50%
**2**	3,136	6.22%	3,033	6.01%		698	5.61%	677	5.44%		2,369	6.33%	2,338	6.25%
**3+**	46,155	91.51%	46,063	91.33%		11,499	92.47%	11,529	92.71%		34,145	91.28%	34,090	91.14%
Congestive heart failure*	27,590	54.70%	27,606	54.74%		6,658	53.54%	6,659	53.55%		19,715	52.71%	19,644	52.52%
Diabetes mellitus*	24,402	48.38%	24,348	48.28%		6,016	48.38%	5,938	47.75%		17,874	47.79%	17,921	47.91%
Hypertension*	47,544	94.27%	47,478	94.14%		11,818	95.03%	11,845	95.25%		35,237	94.20%	35,199	94.10%
Renal disease*	24,665	48.90%	24,761	49.09%		5,087	40.91%	5,081	40.86%		15,743	42.09%	15,702	41.98%
Liver disease*	4,996	9.91%	5,086	10.08%		1,123	9.03%	1,119	9.00%		3,543	9.47%	3,534	9.45%
Myocardial infarction*	11,369	22.54%	11,327	22.46%		2,420	19.46%	2,513	20.21%		7,882	21.07%	7,877	21.06%
Dyspepsia or stomach discomfort*	17,443	34.59%	17,466	34.63%		4,524	36.38%	4,657	37.45%		13,434	35.91%	13,365	35.73%
Non-stroke/SE peripheral vascular disease*	21,390	42.41%	21,558	42.74%		4,867	39.14%	4,972	39.98%		15,384	41.13%	15,440	41.28%
Stroke/SE*	13,826	27.41%	13,885	27.53%		3,401	27.35%	3,421	27.51%		9,413	25.17%	9,472	25.32%
Transient ischemic attack*	11,098	22.00%	11,152	22.11%		2,234	17.96%	2,205	17.73%		6,695	17.90%	6,712	17.94%
Anemia and coagulation defects*	36,482	72.33%	36,490	72.35%		9,145	73.54%	9,166	73.71%		27,348	73.11%	27,289	72.96%
Alcoholism*	1,333	2.64%	1,297	2.57%		266	2.14%	249	2.00%		975	2.61%	1,000	2.67%
**Peripheral artery disease**	20,260	40.17%	21,154	41.94%		4,762	38.29%	4,894	39.35%		14,941	39.94%	15,181	40.59%
**Coronary artery disease**	31,712	62.88%	31,993	63.43%		7,814	62.83%	7,872	63.30%		23,197	62.02%	23,320	62.34%
Baseline Medication Use*														
**ACE/ARB**	30,344	60.16%	30,180	59.84%		7,679	61.75%	7,629	61.35%		22,791	60.93%	22,705	60.70%
**Amiodarone**	8,159	16.18%	8,172	16.20%		1,664	13.38%	1,650	13.27%		5,615	15.01%	5,696	15.23%
**Beta blockers**	30,802	61.07%	30,966	61.40%		7,412	59.60%	7,404	59.54%		22,357	59.77%	22,278	59.56%
**H2-receptor antagonists**	5,622	11.15%	5,630	11.16%		1,236	9.94%	1,248	10.04%		4,129	11.04%	4,125	11.03%
**Proton pump inhibitors**	27,503	54.53%	27,526	54.58%		6,911	55.57%	6,973	56.07%		20,502	54.81%	20,488	54.77%
**Statins**	34,152	67.71%	34,139	67.69%		8,270	66.50%	8,372	67.32%		24,639	65.87%	24,624	65.83%
**Anti-platelets**	10,443	20.71%	10,371	20.56%		2,087	16.78%	2,154	17.32%		7,313	19.55%	7,135	19.07%
**NSAIDs**	10,330	20.48%	10,258	20.34%		2,663	21.41%	2,651	21.32%		8,202	21.93%	8,135	21.75%
**Dose of the Index Prescription** ^**◊**^														
**Standard Dose** ^‡^	30,645	60.76%				8,503	68.37%				21,593	57.73%		
**Lower Dose** ^§^	19,824	39.31%				3,938	31.67%				15,962	42.67%		
Prior OAC Utilization*														
**Patients without an OAC claim ≥ 12 months before prior bleed date**	21,909	43.44%	21,930	43.48%		3,245	26.09%	3,228	25.96%		14,108	37.72%	14,055	37.58%
**Patients with at least 1 OAC claim ≥ 12 months before prior bleed date**	28,526	56.56%	28,505	56.52%		9,191	73.91%	9,208	74.04%		23,297	62.28%	23,350	62.42%
Type of Prior Bleed*														
**Prior ICH Bleed**	7,010	13.90%	7,017	13.91%		1,592	12.80%	1,593	12.81%		4,381	11.71%	4,419	11.81%
**Prior GI Bleed**	30,668	60.81%	30,712	60.89%		7,811	62.81%	7,856	63.17%		22,729	60.76%	22,785	60.91%
**Prior Other Bleed**	12,757	25.29%	12,706	25.19%		3,033	24.39%	2,987	24.02%		10,295	27.52%	10,201	27.27%
Timing of Bleed*														
**Patients With a Bleed Prior To Treatment Episode**	44,598	88.43%	44,640	88.51%		10,332	83.08%	10,377	83.44%		30,865	82.52%	30,886	82.57%
**Patients With a Bleed During Treatment Episode**	5,837	11.57%	5,795	11.49%		2,104	16.92%	2,059	16.56%		6,540	17.48%	6,519	17.43%
**Time from Prior Bleed to Index date (in days)**	430.42	489.45	391.48	425.78		310.78	386.37	315.18	379.39		373.46	444.72	353.19	406.73
**Patients with ≥ 1 year between bleed and treatment**	29,585	58.66%	30,148	59.78%		8,729	70.19%	8,551	68.76%		23,606	63.11%	23,871	63.82%
**Patients with < 1 year between bleed and treatment**	20,850	41.34%	20,287	40.22%		3,707	29.81%	3,885	31.24%		13,799	36.89%	13,534	36.18%
**Follow-up Time (in days)**	235.1	248.9	251.2	275.1		276.3	312.7	267.8	289.4		249.9	285.5	260.3	284.2
**Median**	138		144			154		157			135		149	

ACE/ARB: angiotensin converting enzyme inhibitors/angiotensin-receptor blockers; GI: gastrointestinal; ICH: intracranial hemorrhage; NOAC: non-vitamin K antagonist oral anticoagulants; NSAID: non-steroidal anti-inflammatory; OAC: oral anticoagulant; SD: standard deviation; SE: systemic embolism; CHA_2_DS_2_VASC: congestive heart failure, hypertension, age ≥ 75 years, diabetes mellitus, prior stroke or transient ischemic attack or thromboembolism, vascular disease, age 65–74 years, sex category; HAS-BLED: hypertension, abnormal renal or liver function, stroke, bleeding, labile INRs (international normalized ratio), elderly, and drugs or alcohol

* Variables controlled for in the propensity score matching

^†^ as the INR value is not available in the databases, a modified HAS-BLED score was calculated with a range of 0 to 8

◊ Patients could overlap on drugs at index and be in both dosing categories

^‡^ Standard dose: 5 mg Apixaban, 150 mg Dabigatran, 20 mg Rivaroxaban

^§^ Lower dose: 2.5 mg Apixaban, 75 or 110 mg Dabigatran, 10 or 15 mg Rivaroxaban. 69 patients received 110 mg dabigatran in the dabigatran-warfarin cohort, 2,786 patients received 10 mg rivaroxaban in the rivaroxaban-warfarin cohort

**Table 2:** Baseline Characteristics of Patients Prescribed NOACs vs. NOACs after Propensity Score Matching

	Apixaban Cohort (N = 12,275)		Dabigatran Cohort (N = 12,275)			Apixaban Cohort (N = 35,376)		Rivaroxaban Cohort (N = 35,376)			Dabigatran Cohort (N = 12,297)		Rivaroxaban Cohort (N = 12,297)	
	**N/** **Mean**	**%/** **SD**	**N/** **Mean**	**%/** **SD**		**N/** **Mean**	**%/** **SD**	**N/** **Mean**	**%/** **SD**		**N/** **Mean**	**%/** **SD**	**N/** **Mean**	**%/** **SD**
Age*	77.52	8.65	77.59	8.54		77.40	8.97	77.31	9.05		77.59	8.53	77.41	8.70
**18–54**	119	0.97%	106	0.86%		388	1.10%	448	1.27%		107	0.87%	143	1.16%
**55–64**	404	3.29%	419	3.41%		1,375	3.89%	1,415	4.00%		409	3.33%	413	3.36%
**65–74**	3,916	31.90%	3,865	31.49%		11,393	32.21%	11,223	31.72%		3,892	31.65%	3,869	31.46%
**75–79**	2,790	22.73%	2,802	22.83%		7,839	22.16%	7,923	22.40%		2,793	22.71%	2,804	22.80%
**≥ 80**	5,046	41.11%	5,083	41.41%		14,381	40.65%	14,367	40.61%		5,096	41.44%	5,068	41.21%
Gender*														
**Male**	6,079	49.52%	6,123	49.88%		17,047	48.19%	17,076	48.27%		6,161	50.10%	6,253	50.85%
**Female**	6,196	50.48%	6,152	50.12%		18,329	51.81%	18,300	51.73%		6,136	49.90%	6,044	49.15%
**Baseline Comorbidity**														
Deyo-Charlson Comorbidity Index*	4.82	3.12	4.84	3.14		5.01	3.25	5.00	3.24		4.82	3.14	4.80	3.15
**CHA**_**2**_ **DS**_**2**_ **-VASc Score**	5.24	1.73	5.33	1.77		5.22	1.76	5.26	1.79		5.32	1.77	5.26	1.78
**0**	15	0.12%	14	0.11%		55	0.16%	70	0.20%		16	0.13%	26	0.21%
**1**	135	1.10%	142	1.16%		462	1.31%	447	1.26%		146	1.19%	145	1.18%
**2**	520	4.24%	520	4.24%		1,651	4.67%	1,655	4.68%		535	4.35%	544	4.42%
**3**	1,284	10.46%	1,221	9.95%		3,782	10.69%	3,788	10.71%		1,234	10.03%	1,343	10.92%
**4+**	10,321	84.08%	10,378	84.55%		29,426	83.18%	29,416	83.15%		10,366	84.30%	10,239	83.26%
**HAS-BLED Score** ^†^	4.12	1.15	4.16	1.16		4.12	1.20	4.14	1.21		4.15	1.16	4.12	1.17
**0**	4	0.03%	6	0.05%		33	0.09%	35	0.10%		6	0.05%	8	0.07%
**1**	226	1.84%	228	1.86%		799	2.26%	816	2.31%		231	1.88%	248	2.02%
**2**	684	5.57%	686	5.59%		2,284	6.46%	2,275	6.43%		687	5.59%	706	5.74%
**3+**	11,361	92.55%	11,355	92.51%		32,260	91.19%	32,250	91.16%		11,373	92.49%	11,335	92.18%
Congestive heart failure*	6,624	53.96%	6,590	53.69%		18,608	52.60%	18,635	52.68%		6,567	53.40%	6,552	53.28%
Diabetes mellitus*	5,908	48.13%	5,930	48.31%		16,800	47.49%	16,837	47.59%		5,930	48.22%	5,880	47.82%
Hypertension*	11,663	95.01%	11,670	95.07%		33,326	94.21%	33,338	94.24%		11,684	95.02%	11,652	94.75%
Renal disease*	5,032	40.99%	5,077	41.36%		15,404	43.54%	15,339	43.36%		5,036	40.95%	4,990	40.58%
Liver disease*	1,145	9.33%	1,119	9.12%		3,374	9.54%	3,397	9.60%		1,115	9.07%	1,114	9.06%
Myocardial infarction*	2,366	19.27%	2,406	19.60%		7,564	21.38%	7,574	21.41%		2,403	19.54%	2,435	19.80%
Dyspepsia or stomach discomfort*	4,465	36.37%	4,468	36.40%		12,608	35.64%	12,515	35.38%		4,478	36.42%	4,344	35.33%
Non-stroke/SE peripheral vascular disease*	4,790	39.02%	4,845	39.47%		14,628	41.35%	14,610	41.30%		4,843	39.38%	4,823	39.22%
Stroke/SE*	3,306	26.93%	3,357	27.35%		9,060	25.61%	9,070	25.64%		3,323	27.02%	3,303	26.86%
Transient ischemic attack*	2,261	18.42%	2,238	18.23%		6,699	18.94%	6,696	18.93%		2,217	18.03%	2,208	17.96%
Anemia and coagulation defects*	9,015	73.44%	9,035	73.60%		25,487	72.05%	25,521	72.14%		9,056	73.64%	9,086	73.89%
Alcoholism*	250	2.04%	257	2.09%		1,001	2.83%	1,002	2.83%		259	2.11%	264	2.15%
**Peripheral artery disease**	4,536	36.95%	4,739	38.61%		13,843	39.13%	14,184	40.09%		4,735	38.51%	4,711	38.31%
**Coronary artery disease**	7,568	61.65%	7,731	62.98%		21,916	61.95%	22,018	62.24%		7,730	62.86%	7,732	62.88%
Baseline Medication Use*														
**ACE/ARB**	7,549	61.50%	7,571	61.68%		21,570	60.97%	21,508	60.80%		7,586	61.69%	7,645	62.17%
**Amiodarone**	1,633	13.30%	1,652	13.46%		5,420	15.32%	5,481	15.49%		1,653	13.44%	1,623	13.20%
**Beta blockers**	7,352	59.89%	7,337	59.77%		21,295	60.20%	21,324	60.28%		7,322	59.64%	7,355	59.81%
**H2-receptor antagonists**	1,242	10.12%	1,232	10.04%		3,945	11.15%	3,929	11.11%		1,230	10.00%	1,194	9.71%
**Proton pump inhibitors**	6,787	55.29%	6,833	55.67%		19,358	54.72%	19,441	54.96%		6,854	55.74%	6,847	55.68%
**Statins**	8,132	66.25%	8,170	66.56%		23,482	66.38%	23,436	66.25%		8,168	66.42%	8,194	66.63%
**Anti-platelets**	2,105	17.15%	2,085	16.99%		7,010	19.82%	7,106	20.09%		2,069	16.83%	2,075	16.87%
**NSAIDs**	2,686	21.88%	2,646	21.56%		7,824	22.12%	7,854	22.20%		2,659	21.62%	2,714	22.07%
**Dose of the Index Prescription** ^**◊**^														
**Standard Dose** ^‡^	7,623	62.10%	8,371	68.20%		22,082	62.42%	20,164	57.00%		8,402	68.33%	7,114	57.85%
**Lower Dose** ^§^	4,659	37.96%	3,909	31.85%		13,320	37.65%	15,359	43.42%		3,900	31.72%	5,227	42.51%
Prior OAC Utilization*														
**Patients without an OAC claim 12 months before prior bleed date**	3,242	26.41%	3,261	26.57%		14,426	40.78%	14,463	40.88%		3,253	26.45%	3,252	26.45%
**Patients with at least 1 OAC claim 12 months before prior bleed date**	9,033	73.59%	9,014	73.43%		20,950	59.22%	20,913	59.12%		9,044	73.55%	9,045	73.55%
Type of Prior Bleed*														
**Prior ICH Bleed**	1,560	12.71%	1,580	12.87%		4,421	12.50%	4,352	12.30%		1,548	12.59%	1,515	12.32%
**Prior GI Bleed**	7,631	62.17%	7,723	62.92%		21,584	61.01%	21,600	61.06%		7,750	63.02%	7,763	63.13%
**Prior Other Bleed**	3,084	25.12%	2,972	24.21%		9,371	26.49%	9,424	26.64%		2,999	24.39%	3,019	24.55%
Timing of Bleed*														
**Patients With a Bleed Prior To Treatment Episode**	10,237	83.40%	10,235	83.38%		30,335	85.75%	30,354	85.80%		10,196	82.91%	10,227	83.17%
**Patients With a Bleed During Treatment Episode**	2,038	16.60%	2,040	16.62%		5,041	14.25%	5,022	14.20%		2,101	17.09%	2,070	16.83%
**Time from Prior Bleed to Index Date (in days)**	342.67	432.07	313.04	387.44		412.01	482.43	393.35	452.58		312.19	387.48	338.25	417.61
**Patients with ≥ 1 year between bleed and treatment**	8,258	67.27%	8,584	69.93%		21,404	60.50%	21,562	60.95%		8,606	69.98%	8,247	67.07%
**Patients with < 1 year between bleed and treatment**	4,017	32.73%	3,691	30.07%		13,972	39.50%	13,814	39.05%		3,691	30.02%	4,050	32.93%
**Follow-up Time (in days)**	257.3	267.7	278.5	314.5		243.0	254.2	247.8	281.5		279.9	316.7	258.8	287.4
**Median**	152		156			144		133			156		145	

ACE/ARB; angiotensin converting enzyme inhibitors/angiotensin-receptor blockers; GI: gastrointestinal; ICH: intracranial hemorrhage; NOAC: non-vitamin K antagonist oral anticoagulants; NSAID: non-steroidal anti-inflammatory; OAC: oral anticoagulant; SD: standard deviation; SE: systemic embolism; CHA_2_DS_2_VASC: congestive heart failure, hypertension, age ≥ 75 years, diabetes mellitus, prior stroke or transient ischemic attack or thromboembolism, vascular disease, age 65–74 years, sex category; HAS-BLED: hypertension, abnormal renal or liver function, stroke, bleeding, labile INRs (international normalized ratio), elderly, and drugs or alcohol

* Variables controlled for in the propensity score matching

^†^ as the INR value is not available in the databases, a modified HAS-BLED score was calculated with a range of 0 to 8

◊ Patients could overlap on drugs at index and be in both dosing categories

^‡^ Standard dose: 5 mg Apixaban, 150 mg Dabigatran, 20 mg Rivaroxaban

^§^ Lower dose: 2.5 mg Apixaban, 75 or 110 mg Dabigatran, 10 or 15 mg Rivaroxaban. 2,706 and 802 patients received 10 mg rivaroxaban in the apixaban-rivaroxaban and dabigatran-rivaroxaban cohort, respectively, while 68 and 69 patients received 110 mg dabigatran in the apixaban-dabigatran and dabigatran-rivaroxaban cohort, respectively

Stroke/SE and MB incidence after index OAC were calculated using the number of events divided by total person-years at risk and multiplied by 100, with Kaplan-Meier curves to illustrate cumulative rates. Cox proportional hazard models with robust sandwich estimates were also applied to the PSM population within the pooled dataset to evaluate the comparative risks.[29] OAC treatment was included as the independent variable in the Cox models because all matched covariates were similar after PSM between the 2 comparative arms. *P*-values of 0.05 were used as the threshold for statistical significance.

### Subgroup Analysis

Three subgroup analyses were conducted. The first two subgroup analyses were two interaction analyses, one between treatment and prior OAC use (with prior OAC use vs. without prior OAC use), and another between treatment and type of prior bleed (i.e., ICH, GI, other). The statistical significance (*p* < 0.10) of the interaction between treatment and prior OAC use or bleed type was evaluated.

The third analysis was the dose subgroup analysis for the NOAC cohorts. Standard-dose (i.e., apixaban 5 mg twice-daily, dabigatran 150 mg twice-daily, rivaroxaban 20 mg daily) and lower-dose (apixaban 2.5 mg twice-daily, dabigatran 75/110 mg twice-daily, rivaroxaban 15 mg/10 mg daily) patients were examined separately based on index prescription dosage. Warfarin cohort patients were matched to NOAC patients with either dosage. INR data was not available for this analysis. The statistical methods of the main analysis were used, wherein 1:1 PSM patients in each dataset were pooled and compared.

### Institutional Review Board approval

Institutional Review Board review and approval were not required because this study did not involve the collection, use, or transmittal of individually identifiable data. Both the datasets and the security of the offices where analysis was completed (and where the datasets are kept) meet the requirements of the Health Insurance Portability and Accountability Act of 1996.

## Results

After applying the selection criteria, a total of 244,563 NVAF patients with prior bleeding events were identified, including 55,094 (22.5%) prescribed apixaban, 12,500 (5.1%) dabigatran, 38,246 (15.6%) rivaroxaban, and 138,723 (56.7%) warfarin^1^. Among patients with a prior bleed, 60.0% had a prior GI bleed, 12.2% had a prior ICH bleed, and 27.9% had a bleed at another key site. Most patients had the bleeding event more than or equal to one year before the index date (65.4%) and had OAC treatment in the 12 months before the bleeding event (67.7%). For apixaban, dabigatran, and rivaroxaban patients, 38.8%, 31.5%, and 42.3% used lower dosage regimens, respectively. The baseline characteristics of patients in each treatment cohort can be found in Supplemental Table 2.

The unadjusted incidence rate of stroke/SE—including ischemic stroke, hemorrhagic stroke, and SE—was 2.7 (apixaban), 2.6 (dabigatran), 2.5 (rivaroxaban), and 2.9 (warfarin) per 100 person-years (data not shown). The unadjusted incidence rate of MB—including GI bleeding, ICH, and other MB—was 9.4 (apixaban), 10.9 (dabigatran), 13.4 (rivaroxaban), and 13.6 (warfarin) per 100 person-years, respectively (data not shown).

After 1:1 PSM, a total of 50,435 apixaban–warfarin, 12,436 dabigatran–warfarin, 37,405 rivaroxaban–warfarin, 12,275 apixaban–dabigatran, 35,376 apixaban–rivaroxaban, and 12,297 dabigatran–rivaroxaban pairs were evaluated. The mean age was 77–78 years for the matched cohorts, and the mean follow-up time was 8–9 months. Complete descriptive baseline characteristics of the pooled analysis are presented in Tables 1 and 2. All baseline variables included in the PSM logistic models were balanced with standardized differences < 10% (Tables 1 and 2).

### NOAC–Warfarin comparisons after PSM

**Fig. 2:**
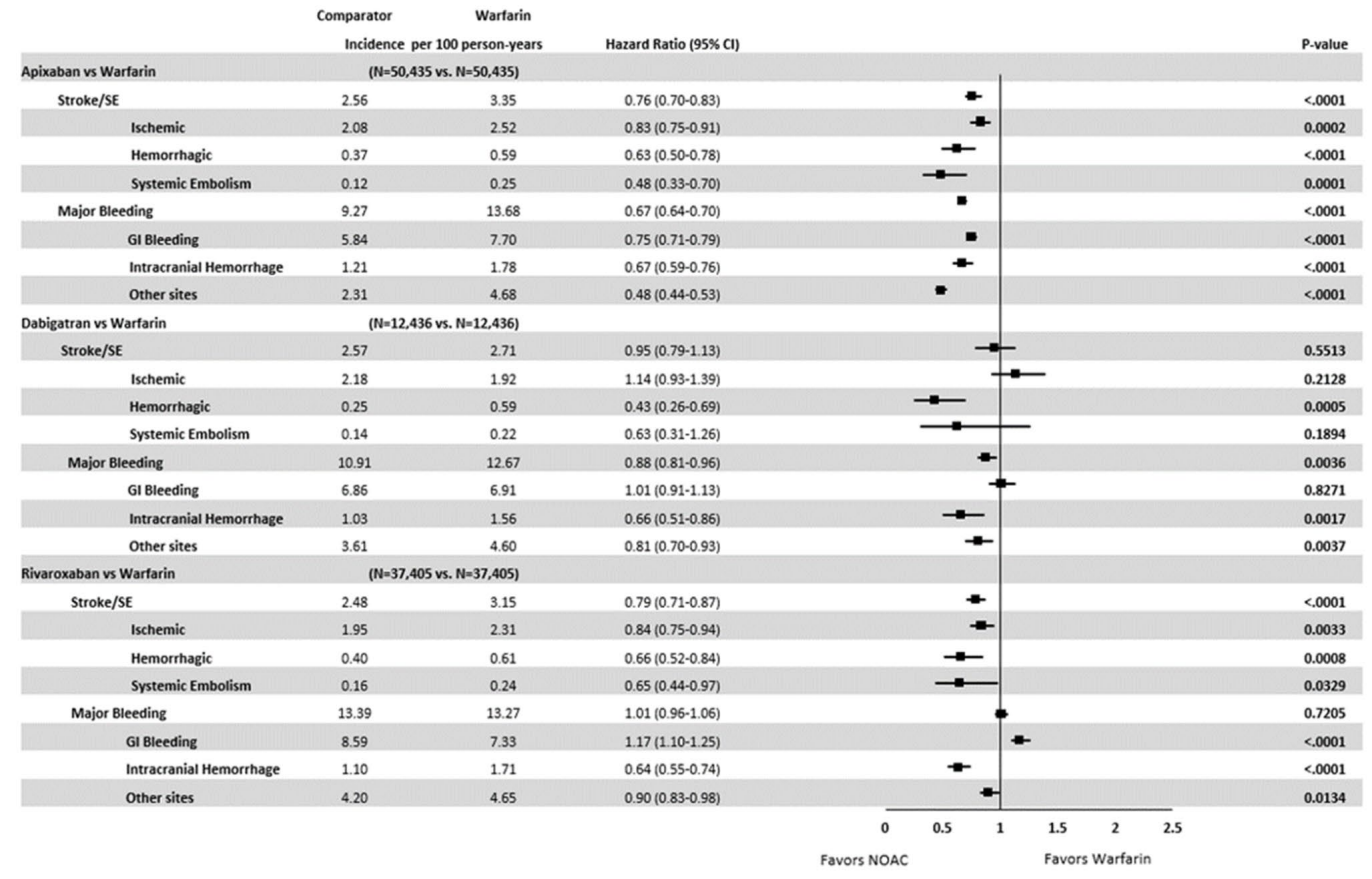
**Propensity Score-Matched Incidence Rates and Hazard Ratios of Stroke/SE and Major Bleeding for NOAC versus Warfarin** Cox proportional hazard models with robust sandwich estimates were used to evaluate the risk of stroke/SE and major bleeding CI: confidence interval; GI: gastrointestinal; ICH: intracranial hemorrhage; NOAC: non-vitamin K antagonist oral anticoagulant; SE: systemic embolism

Among NVAF patients with prior bleed, apixaban (hazard ratio [HR]: 0.76, 95% confidence interval [CI]: 0.70–0.83) and rivaroxaban use (HR: 0.79, 95% CI: 0.71–0.87) were associated with a lower risk of stroke/SE compared with warfarin. Ischemic stroke was the most prevalent type of stroke/SE, with a lower risk in apixaban (HR: 0.83, 95% CI: 0.75–0.91) and rivaroxaban (HR: 0.84, 95% CI: 0.75–0.94) patients compared with warfarin patients. (Fig. 2).

Apixaban (HR: 0.67, 95% CI: 0.64–0.70) and dabigatran (HR: 0.88, 95% CI: 0.81–0.96) were associated with a lower risk of MB compared with warfarin. Apixaban was associated with a lower risk (HR:0.75, 95% CI: 0.71–0.79), and rivaroxaban was associated with a higher risk (HR: 1.17, 95% CI:1.10–1.25) of GI bleeding (the most prevalent type of MB) vs. warfarin. All NOACs were associated with a lower risk of ICH vs. warfarin (apixaban: HR: 0.67, 95% CI: 0.59–0.76; dabigatran: HR: 0.66, 95% CI: 0.51–0.86; rivaroxaban: HR: 0.64, 95% CI: 0.55–0.74). (Fig. 2).

### NOAC–NOAC comparisons after PSM

**Fig. 3:**
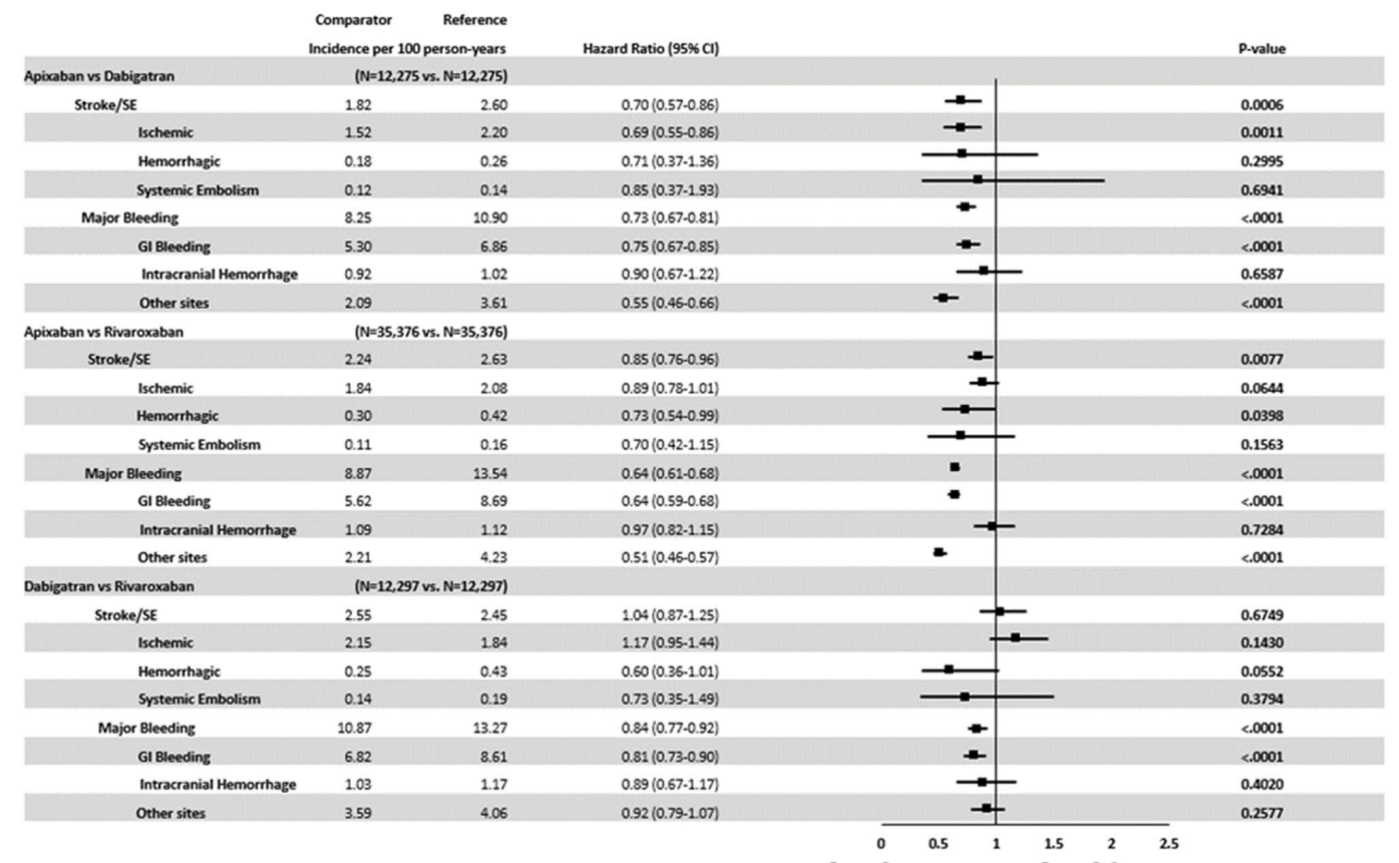
**Propensity Score-Matched Incidence Rates and Hazard Ratios of Stroke/SE and Major Bleeding for NOAC Comparisons** Cox proportional hazard models with robust sandwich estimates were used to evaluate the risk of stroke/SE and major bleeding CI: confidence interval; GI: gastrointestinal; ICH: intracranial hemorrhage; NOAC: non-vitamin K antagonist oral anticoagulant; SE: systemic embolism

Apixaban patients had a lower risk of stroke/SE compared with dabigatran (HR: 0.70, 95% CI: 0.57–0.86) and rivaroxaban (HR: 0.85, 95% CI: 0.76–0.96), and dabigatran patients were associated with a similar risk of stroke/SE compared with rivaroxaban (HR: 1.04, 95% CI: 0.87–1.25) (Fig. 3). Compared with dabigatran and rivaroxaban, apixaban was associated with a lower risk of MB (dabigatran: HR: 0.73, 95% CI: 0.67–0.81, rivaroxaban: HR: 0.64, 95% CI: 0.61–0.68) and lower risk of GI bleeding (dabigatran HR: 0.75, 95% CI: 0.67–0.85 and rivaroxaban HR: 0.64, 95% CI: 0.59–0.68). Compared with rivaroxaban, dabigatran was associated with a lower risk of MB and GI bleeding (MB HR: 0.84, 95% CI: 0.77–0.92 and GI HR: 0.81, 95% CI: 0.73–0.90) (Fig. 3).

The Kaplan-Meier curves for cumulative incidence of stroke/SE and MB in the matched populations appear in Supplemental Figs. 1 and 2.

### Subgroup analyses

In the first interaction analysis of treatment with prior OAC use, treatment effects were generally consistent regardless of prior OAC use. For dabigatran vs. warfarin and dabigatran vs. rivaroxaban, patients without prior OAC use experienced a greater magnitude of reduction in the risk of MB compared with patients with prior OAC use. Additionally, for apixaban vs. dabigatran, a similar risk of MB was observed among patients without prior OAC use while a lower risk of MB was found in those with prior OAC use (Supplemental Tables 3 and 4). No significant interactions were observed for treatment and type of prior bleed for stroke/SE or MB (Supplemental Tables 5 and 6).

Results of the dose subgroup analysis were generally consistent with the main analysis; however, the risk of stroke/SE was similar between standard-dose apixaban when compared with standard-dose rivaroxaban (Supplemental Table 7). Among patients with low-dose rivaroxaban, the risk of stroke/SE was similar compared with warfarin. There was no significant differences for stroke/SE between the low-dose NOACs [i.e. apixaban vs. rivaroxaban and apixaban vs. dabigatran] (Supplemental Table 8). Also, there was no significant difference in the risk of MB when comparing low dose dabigatran to rivaroxaban (Supplemental Table 8).

## Discussion

To our knowledge, this is one of the first retrospective, real-world cohort analyses among U.S. patients to compare individual NOACs to warfarin and to one another in a large sample of NVAF patients with previous bleeding. Leveraging data from 5 large U.S. claims databases, this study found that apixaban and rivaroxaban were associated with a lower risk of stroke/SE, and dabigatran was associated with a similar risk of stroke/SE, when compared with warfarin. Apixaban and dabigatran were associated with a lower risk of MB, and rivaroxaban was associated with a similar risk of MB, compared with warfarin. Subgroup analyses of prior OAC use, type of prior bleed and NOAC dose showed generally consistent findings to the main analysis.

The current findings are consistent with published studies reporting favorable outcomes on stroke/SE and/or MB for NOACs vs. warfarin in AF patients with prior major hemorrhage.[22, 23, 30–32] Most of these studies used datasets from Danish, Korean, or Taiwanese populations, which may limit the generalizability of findings to U.S. patients. For example, Lee et al.[32] found NOACs were associated with multiple positive outcomes compared with warfarin in AF patients with previous ICH, including a lower risk of fatal and nonfatal ischemic stroke, ICH, the composite outcome of stroke plus ICH, death from the composite outcome, and all-cause mortality. Kwon et al.[31] similarly observed significantly lower rates of fatal and nonfatal ischemic stroke, fatal and nonfatal ICH, nonfatal GI bleed, and all-cause death with NOACs vs. warfarin in AF patients with a prior GI bleed.

The current study extends the findings from the existing evidence by comparing each NOAC individually against warfarin and against one another and using a large U.S. cohort that includes multiple types of bleeding (i.e., ICH, GI, and other MB). Our findings suggest NOACs may represent a safe and effective option for initiating or resuming anticoagulation in AF patients with prior bleeding, and that, compared with warfarin, these drugs could offer at least comparable— and in some cases possibly better—protection against stroke/SE and MB. However, these findings need to be confirmed by randomized controlled trials in AF patients with a history of ICH, GI bleed, or other MB. Some ongoing and recently completed randomized clinical trials will provide more insights about the effects of NOACs on thromboembolic and bleeding events in AF patients with a history of ICH (NCT03996772 and NCT02998905).

Across different NOACs, apixaban was associated with a lower risk of stroke/SE and MB compared with dabigatran and rivaroxaban, and dabigatran was associated with lower risk of MB than rivaroxaban. Our findings were consistent with Kwon et al.[31] who reported a lower risk of MB with apixaban vs. dabigatran, rivaroxaban, and edoxaban. Nonetheless, only head-to-head clinical trials will provide definitive answers about the efficacy and safety of NOACs vs. NOACs in the AF population, and in AF patients with a history of bleeding specifically.

The effectiveness and safety of different NOACs have not been previously established in a U.S. cohort of NVAF patients with prior bleeding. This represents a major literature gap, given that NOAC prescribing in the United States and Europe has increased considerably over the past decade,[33–35] with the American College of Cardiology, American Heart Association, and European Society of Cardiology now recommending NOACs over warfarin to reduce stroke risk in AF populations.[36–38] Formal clinical practice guidelines are still lacking as to which NOAC to prescribe, when, and at what dose for AF patients with prior bleeding. In response to growing evidence about the benefits of NOACs, cardiologists have expressed a desire for more data to guide them in making prescribing decisions—namely, more real-world data rather than just clinical trial findings, and more data comparing NOACs to one another rather than to warfarin only.[39] The current analysis of NOACs vs. warfarin and NOACs vs. NOACs in a large US cohort of NVAF patients with prior bleeding could be useful to help inform clinical decision-making in this challenging patient population.

### Limitations

Our findings should be interpreted in the context of a few limitations. As is the case with all retrospective observational studies, causal relationships cannot be determined between the study variables and outcomes of interest. The datasets analyzed in this study were limited to an extent, which could affect results: potential residual confounders, such as over-the-counter aspirin use, serum creatinine/creatinine clearance, and laboratory values, were unavailable, and their absence could introduce bias. Given that ICD, CPT, and HCPCS codes were used to identify the diagnoses and procedures, some variables in the datasets may lack clinical accuracy due to human data entry errors. Finally, the lack of laboratory information (e.g., lack of INR to determine time in therapeutic range) makes it difficult to assess the quality of warfarin control. Nevertheless, by including patients with potentially poor quality of warfarin treatment, this study may reflect real-world clinical practice.[40] It should also be noted that unobserved heterogeneity may exist across the 5 datasets used in this analysis. For the commercial datasets, although some of them include data from different insurance plans that do not overlap at the plan level, others are employer-based claims datasets which may contain duplicate patient records when pooled together. But the likelihood of duplicate observations is relatively low, researched to be 0.5%, and is not likely to have a significant impact on study results.[41] To avoid potential duplications the commercial datasets with Medicare data, patients with Medicare supplemental plans in MarketScan and PharMetrics data were excluded. For Optum and Humana data, beneficiaries aged ≥ 65 years are not covered in Medicare data and therefore do not have duplicates.

## Conclusions

To our knowledge, this is the first real-world data analysis of stroke/SE and MB outcomes of NOACs vs. warfarin and vs. one another in a U.S. sample of NVAF patients with prior bleeding. The results indicate that treatment with NOACs was associated with similar or lower risk of stroke/SE and MB compared with warfarin and variable risk of stroke/SE and MB against each other.

## Electronic supplementary material

Below is the link to the electronic supplementary material.

Supplementary Material 1

## Footnotes

1 There were 241 patients with an edoxaban treatment episode captured, which accounted for 0.1% of the sample.
