# Supplementary material for: Effectiveness and safety of oral anticoagulants in non-valvular atrial fibrillation patients with prior bleeding events: a retrospective analysis of administrative claims databases
Source: J Thromb Thrombolysis. 2022 May 17;54(1):33–46. doi: 10.1007/s11239-022-02660-2 (PMC9259524; doi:10.1007/s11239-022-02660-2)
Supplement: Supplementary file 1 — Supplementary Material 1 [file 11239_2022_2660_MOESM1_ESM.docx]

**Supplemental Table 1. Codes used for identifying outcomes**

| **Diagnosis** | **ICD-9-CM Diagnosis and Procedure Codes** | **ICD-10-CM Diagnosis and Procedure Codes** |
| --- | --- | --- |
| Hemorrhagic Stroke | 430.xx-432.xx | I6000, I6001, I6002, I6010, I6011, I6012, I602, I6030, I6031, I6032, I604, I6050, I6051, I6052, I606, I607, I608, I609, I610, I611, I612, I613, I614, I615, I616, I618, I619 |
|  | Cases were excluded if traumatic brain injury (ICD-9-CM: 800-804, 850-854) was present during hospitalization. |  |
| Ischemic Stroke | 433.x1, 434.x1, 436 | I6300, I63011, I63012, I63013, I63019, I6302, I63031, I63032, I63033, I63039, I6309, I6310, I63111, I63112, I63113, I63119, I6312, I63131, I63132, I63133, I63139, I6319, I6320, I63211, I63212, I63213, I63219, I6322, I63231, I63232, I63233, I63239, I6329, I6330, I63311, I63312, I63313, I63319, I63321, I63322, I63323, I63329, I63331, I63332, I63333, I63339, I63341, I63342, I63343, I63349, I6339, I6340, I63411, I63412, I63413, I63419, I63421, I63422, I63423, I63429, I63431, I63432, I63433, I63439, I63441, I63442, I63443, I63449, I6349, I6350, I63511, I63512, I63513, I63519, I63521, I63522, I63523, I63529, I63531, I63532, I63533, I63539, I63541, I63542, I63543, I63549, I6359, I636, I638, I639, I6789 |
| Systemic Embolism | 444.x, 445.x | I7401, I7409, I7410, I7411, I7419, I742, I743, I744, I745, I748, I749, I75011, I75012, I75013, I75019, I75021, I75022, I75023, I75029, I7581, I7589 |
| Major Gastrointestinal Bleeding | 456.0, 456.20, 530.82, 531.0x, 531.2x, 531.4x, 531.6x, 532.0x, 532.2x, 532.4x, 532.6x, 533.0x, 533.2x, 533.4x, 533.6x, 534.0x, 534.2x, 534.4x, 534.6x, 535.01, 535.11, 535.21, 535.31, 535.41, 535.51, 535.61, 537.83, 562.02, 562.03, 562.12, 562.13, 568.81, 569.3, 569.85, 578.x | I8501, I8511, K2211, K226, K250, K252, K254, K256, K260, K262, K264, K266, K270, K272, K274, K276, K280, K282, K284, K286, K2901, K2921, K2931, K2941, K2951, K2961, K2971, K2981, K2991, K31811, K3182, K5521, K5701, K5711, K5713, K5721, K5731, K5733, K5741, K5751, K5753, K5781, K5791, K5793, K625, K6381, K661, K920, K921, K922, K9161, K9162, K91840, K91841 |
|  | Procedure code: 44.43 |  |
| Major Intracranial Hemorrhage | 430, 431, 432.0, 432.1, 432.9, 852.0x, 852.2x, 852.4x, 853.0x, | I6000, I6001, I6002, I6010, I6011, I6012, I602, I6030, I6031, I6032, I604, I6050, I6051, I6052, I606, I607, I608, I609, I610, I611, I612, I613, I614, I615, I616, I618, I619, I6200, I6201, I6202, I6203, I621, I629, S06340A, S06341A, S06342A, S06343A, S06344A, S06345A, S06346A, S06347A, S06348A, S06349A, S06350A, S06351A, S06352A, S06353A, S06354A, S06355A, S06356A, S06357A, S06358A, S06359A, S06360A, S06361A, S06362A, S06363A, S06364A, S06365A, S06366A, S06367A, S06368A, S06369A, S064X0A, S064X1A, S064X2A, S064X3A, S064X4A, S064X5A, S064X6A, S064X7A, S064X8A, S064X9A, S065X0A, S065X1A, S065X2A, S065X3A, S065X4A, S065X5A, S065X6A, S065X7A, S065X8A, S065X9A, S066X0A, S066X1A, S066X2A, S066X3A, S066X4A, S066X5A, S066X6A, S066X7A, S066X8A, S066X9A |
| Major Other Hemorrhage | 285.1, 360.43, 362.43, 362.81, 363.61, 363.62, 363.72, 364.41, 372.72, 374.81, 376.32, 377.42, 379.23, 423.0x, 596.7x, 599.7x, 602.1x, 620.1, 621.4, 626.2, 626.5, 626.7, 626.8, 626.9, 719.1x, 782.7, 784.7, 784.8, 786.3x, 958.2, 997.02, 998.11 | D62, D7801, D7802, D7821, D7822, E3601, E3602, E89810, E89811, G9731, G9732, G9751, G9752, H05231, H05232, H05233, H05239, H1130, H1131, H1132, H1133, H2100, H2101, H2102, H2103, H31301, H31302, H31303, H31309, H31311, H31312, H31313, H31319, H31411, H31412, H31413, H31419, H3560, H3561, H3562, H3563, H35731, H35732, H35733, H35739, H4310, H4311, H4312, H4313, H44811, H44812, H44813, H44819, H47021, H47022, H47023, H47029, H59111, H59112, H59113, H59119, H59121, H59122, H59123, H59129, H59311, H59312, H59313, H59319, H59321, H59322, H59323, H59329, H9521, H9522, H9541, H9542, I312, I97410, I97411, I97418, I9742, I97610, I97611, I97618, I97620, J9561, J9562, J95830, J95831, L7601, L7602, L7621, L7622, M2500, M25011, M25012, M25019, M25021, M25022, M25029, M25031, M25032, M25039, M25041, M25042, M25049, M25051, M25052, M25059, M25061, M25062, M25069, M25071, M25072, M25073, M25074, M25075, M25076, M2508, M96810, M96811, M96830, M96831, N421, N857, N897, N920, N923, N930, N938, N939, N9961, N9962, N99820, N99821, R040, R041, R042, R0489, R049, R233, R310, R319, R58, T792XXA; |
|  | Procedure code: 99.04 | 30230N1, 30230P1, 30233N1, 30233P1, 30240N1, 30240P1, 30243N1, 30243P1, 30250N1, 30250P1, 30253N1, 30253P1, 30260N1, 30260P1, 30263N1, 30263P1 |

**Supplemental Table 2. Baseline Characteristics of Patients Prescribed Warfarin, Apixaban, Dabigatran, or Rivaroxaban among Patients with a Prior Bleed**

|  | **Warfarin Cohort (Reference)** | | **Apixaban Cohort** | | **Dabigatran Cohort** | | **Rivaroxaban Cohort** | |
| --- | --- | --- | --- | --- | --- | --- | --- | --- |
|  | **N/Mean** | **%/SD** | **N/Mean** | **%/SD** | **N/Mean** | **%/SD** | **N/Mean** | **%/SD** |
| **Sample Size** | 138,723 |  | 55,094 |  | 12,500 |  | 38,246 |  |
| **Age** | 77.98 | 8.72 | 77.63 | 9.31 | 77.48 | 8.63 | 77.00 | 9.22 |
| **18-54** | 1,107 | 0.80% | 661 | 1.20% | 121 | 0.97% | 585.00 | 1.53% |
| **55-64** | 4,021 | 2.90% | 2,482 | 4.51% | 468 | 3.74% | 1813.00 | 4.74% |
| **65-74** | 42,621 | 30.72% | 16,676 | 30.27% | 3,929 | 31.43% | 12,213 | 31.93% |
| **75-79** | 31,213 | 22.50% | 12,050 | 21.87% | 2,850 | 22.80% | 8,517 | 22.27% |
| **≥80** | 59,761 | 43.08% | 23,225 | 42.16% | 5,132 | 41.06% | 15,118 | 39.53% |
| **Gender** |  |  |  |  |  |  |  |  |
| **Male** | 71,560 | 51.58% | 26,469 | 48.04% | 6,307 | 50.46% | 18,634 | 48.72% |
| **Female** | 67,163 | 48.42% | 28,625 | 51.96% | 6,193 | 49.54% | 19,612 | 51.28% |
| **U.S. Geographic Region** |  |  |  |  |  |  |  |  |
| **Northeast** | 29,268 | 21.10% | 9,375 | 17.02% | 2,589 | 20.71% | 6,704 | 17.53% |
| **Midwest** | 42,215 | 30.43% | 12,307 | 22.34% | 2,771 | 22.17% | 9,050 | 23.66% |
| **South** | 48,337 | 34.84% | 25,790 | 46.81% | 5,236 | 41.89% | 16,693 | 43.65% |
| **West** | 18,691 | 13.47% | 7,536 | 13.68% | 1,886 | 15.09% | 5,714 | 14.94% |
| **Other** | 212 | 0.15% | 86 | 0.16% | 18 | 0.14% | 85 | 0.22% |
| **Baseline Comorbidity** |  |  |  |  |  |  |  |  |
| **Deyo-Charlson Comorbidity Index** | 5.14 | 3.22 | 5.30 | 3.33 | 4.80 | 3.14 | 4.90 | 3.23 |
| **CHA_2_DS_2_-VASc Score** | 5.42 | 1.74 | 5.31 | 1.77 | 5.30 | 1.78 | 5.20 | 1.81 |
| **0** | 114 | 0.08% | 82 | 0.15% | 17 | 0.14% | 98 | 0.26% |
| **1** | 1,277 | 0.92% | 680 | 1.23% | 156 | 1.25% | 583 | 1.52% |
| **2** | 4,927 | 3.55% | 2,402 | 4.36% | 556 | 4.45% | 1,934 | 5.06% |
| **3** | 12,752 | 9.19% | 5,467 | 9.92% | 1,264 | 10.11% | 4,263 | 11.15% |
| **4+** | 119,653 | 86.25% | 46,463 | 84.33% | 10,507 | 84.06% | 31,368 | 82.02% |
| **HAS-BLED Score** | 4.17 | 1.16 | 4.20 | 1.22 | 4.14 | 1.16 | 4.11 | 1.21 |
| **0** | 92 | 0.07% | 50.00 | 0.09% | 7.00 | 0.06% | 50.00 | 0.13% |
| **1** | 2,888 | 2.08% | 1,210 | 2.20% | 234 | 1.87% | 884 | 2.31% |
| **2** | 7,039 | 5.07% | 3,446 | 6.25% | 712 | 5.70% | 2,501 | 6.54% |
| **3+** | 128,704 | 92.78% | 50,388 | 91.46% | 11,547 | 92.38% | 34,811 | 91.02% |
| **Bleeding history** | 110,921 | 79.96% | 38,893 | 70.59% | 10,046 | 80.37% | 28,720 | 75.09% |
| **Congestive Heart Failure (CHF)** | 81,634 | 58.85% | 29,681 | 53.87% | 6,669 | 53.35% | 19,899 | 52.03% |
| **Diabetes Mellitus** | 70,289 | 50.67% | 26,392 | 47.90% | 6,042 | 48.34% | 18,161 | 47.48% |
| **Hypertension** | 130,791 | 94.28% | 52,036 | 94.45% | 11,880 | 95.04% | 36,027 | 94.20% |
| **Renal Disease** | 66,983 | 48.29% | 27,073 | 49.14% | 5,091 | 40.73% | 15,886 | 41.54% |
| **Liver Disease** | 13,044 | 9.40% | 5,541 | 10.06% | 1,131 | 9.05% | 3,665 | 9.58% |
| **Myocardial Infarction** | 30,660 | 22.10% | 12,540 | 22.76% | 2,426 | 19.41% | 8,009 | 20.94% |
| **Dyspepsia or Stomach Discomfort** | 49,497 | 35.68% | 18,954 | 34.40% | 4,552 | 36.42% | 13,730 | 35.90% |
| **Non-stroke/ SE Peripheral vascular disease** | 59,257 | 42.72% | 23,340 | 42.36% | 4,873 | 38.98% | 15,568 | 40.70% |
| **Stroke/SE** | 37,021 | 26.69% | 15,303 | 27.78% | 3,420 | 27.36% | 9,541 | 24.95% |
| **Transient ischemic attack (TIA)** | 21,508 | 15.50% | 12,959 | 23.52% | 2,244 | 17.95% | 6,867 | 17.95% |
| **Anemia and Coagulation Defects** | 108,176 | 77.98% | 39,121 | 71.01% | 9,161 | 73.29% | 27,717 | 72.47% |
| **Alcoholism** | 2,293 | 1.65% | 1,679 | 3.05% | 267 | 2.14% | 1,116 | 2.92% |
| **Peripheral artery disease** | 58,431 | 42.12% | 22,080 | 0.0943207 | 4,767 | 38.14% | 15,116 | 39.52% |
| **Coronary artery disease** | 89,892 | 64.80% | 34,587 | 0.0052586 | 7,846 | 62.77% | 23,617 | 61.75% |
| **Baseline Medication Use** |  |  |  | 0.024859 |  |  |  |  |
| **ACE/ARB** | OB/GYN | 59.42% | 33,280 | 0.0333684 | 7,715 | 61.72% | 23,366 | 61.09% |
| **Amiodarone** | 17,438 | 12.57% | 9,368 | 0.5624821 | 1,678 | 13.42% | 5,799 | 15.16% |
| **Beta blockers** | 81,331 | 58.63% | 33,883 | 0.0528731 | 7,449 | 59.59% | 22,898 | 59.87% |
| **H2-receptor antagonist** | 14,086 | 10.15% | 6,276 | 0.0451764 | 1,240 | 9.92% | 4,216 | 11.02% |
| **Proton pump inhibitor** | 73,919 | 53.29% | 30,046 | 0.1816617 | 6,946 | 55.57% | 21,023 | 54.97% |
| **Statins** | 92,310 | 66.54% | 37,483 | 68.03% | 8,311 | 66.49% | 25,110 | 65.65% |
| **Anti-platelets** | 24,449 | 17.62% | 11,883 | 21.57% | 2,095 | 16.76% | 7,475 | 19.54% |
| **NSAIDS** | 23,246 | 16.76% | 11,857 | 21.52% | 2,698 | 21.58% | 8,625 | 22.55% |
| **Dose of the Index Prescription** |  |  |  |  |  |  |  |  |
| **Standard Dose (5mg Apixaban, 150mg Dabigatran, 20 mg Rivaroxaban )** |  |  | 33,730 | 61.22% | 8,562 | 68.50% | 22,236 | 58.14% |
| **Low Dose (2.5mg Apixaban, 75 mg Dabigatran, 15mg Rivaroxaban)** |  |  | 21,404 | 38.8% | 3,874 | 30.99% | 13,321 | 34.83% |
| **Other Dose (Rivaroxaban 10 mg, Dabigatran 110 mg)** |  |  |  |  | 69 | 0.55% | 2,841 | 7.43% |
| **Prior OAC Utilization** |  |  |  |  |  |  |  |  |
| **Patients without an OAC claim 12 months before prior bleed date** | 34,803 | 25.09% | 26,089 | 47.35% | 3,267 | 26.14% | 14,746 | 38.56% |
| **Patients with at least 1 OAC claim 12 months before prior bleed date** | 103,920 | 74.91% | 29,005 | 52.65% | 9,233 | 73.86% | 23,500 | 61.44% |
| **Type of Most Recent Bleed before index date (prior to or during episode)** |  |  |  |  |  |  |  |  |
| ICH bleed | 15,618 | 11.26% | 8,064 | 14.64% | 1,610 | 12.88% | 4,488 | 11.73% |
| GI bleed | 82,234 | 59.28% | 33,325 | 60.49% | 7,844 | 62.75% | 23,250 | 60.79% |
| Other bleed | 40,871 | 29.46% | 13,705 | 24.88% | 3,046 | 24.37% | 10,508 | 27.47% |
| **Timing of Bleed** |  |  |  |  |  |  |  |  |
| **Patients With a Bleed Prior To Treatment Episode** | 115,120 | 82.99% | 49,143 | 89.20% | 10,393 | 83.14% | 31,599 | 82.62% |
| **Patients With a Bleed During Treatment Episode** | 23,603 | 17.01% | 5,951 | 10.80% | 2,107 | 16.86% | 6,647 | 17.38% |
| **Time from Prior Bleed to Treatment (in days)** | 312.99 | 374.81 | 446.19 | 499.42 | 310.59 | 385.88 | 376.44 | 447.03 |
| **Patients with ≥1 year between bleed and treatment** | 95,557 | 68.88% | 31,525 | 57.22% | 8,774 | 70.19% | 24,013 | 62.79% |
| **Patients with <1 year between bleed and treatment** | 43,166 | 31.12% | 23,569 | 42.78% | 3,726 | 29.81% | 14,233 | 37.21% |

**Supplemental Table 3. Incidence and Hazard Ratios of Stroke/SE and MB among Patients with and without prior OAC use for NOACs vs Warfarin**

| **No prior OAC use** | **Incidence per 100 person-years** | | **Hazard Ratio**  **95% CI** |  | **Prior OAC use** | **Incidence per 100 person-years** | | **Hazard Ratio**  **95% CI** | **Interaction p-value** |
| --- | --- | --- | --- | --- | --- | --- | --- | --- | --- |
|  |  |  |  |  |  |  |  |  |  |
|  | **Apixaban** | **Warfarin** |  |  |  | **Apixaban** | **Warfarin** |  |  |
|  | **(N=21,909)** | **(N=21,930)** |  |  |  | **(N=28,526)** | **(N=28,505)** |  |  |
| Stroke/SE | 2.75 | 3.69 | 0.74 |  | Stroke/SE | 2.43 | 3.12 | 0.78 | 0.6227 |
|  |  |  | (0.65-0.85) |  |  |  |  | (0.69-0.88) |  |
| MB | 8.95 | 13.58 | 0.65 |  | MB | 9.51 | 13.74 | 0.68 | 0.2845 |
|  |  |  | (0.60-0.70) |  |  |  |  | (0.64-0.72) |  |
|  | **Dabigatran** | **Warfarin** |  |  |  | **Dabigatran** | **Warfarin** |  |  |
|  | **(N=3,245)** | **(N=3,228)** |  |  |  | **(N=9,191)** | **(N=9,208)** |  |  |
| Stroke/SE | 2.57 | 3.51 | 0.77 |  | Stroke/SE | 2.57 | 2.47 | 1.03 | 0.1565 |
|  |  |  | (0.54-1.09) |  |  |  |  | (0.84-1.27) |  |
| MB | 7.91 | 12.81 | 0.67 |  | MB | 11.84 | 12.63 | 0.95 | 0.0014 |
|  |  |  | (0.55-0.81) |  |  |  |  | (0.86-1.04) |  |
|  | **Rivaroxaban** | **Warfarin** |  |  |  | **Rivaroxaban** | **Warfarin** |  |  |
|  | **(N=14,108)** | **(N=14,055)** |  |  |  | **(N=23,297)** | **(N=23,350)** |  |  |
| Stroke/SE | 2.54 | 3.31 | 0.76 |  | Stroke/SE | 2.46 | 3.06 | 0.80 | 0.6342 |
|  |  |  | (0.64-0.90) |  |  |  |  | (0.71-0.91) |  |
| MB | 12.91 | 12.55 | 1.02 |  | MB | 13.65 | 13.66 | 1.00 | 0.7402 |
|  |  |  | (0.94-1.11) |  |  |  |  | (0.95-1.06) |  |

**Supplemental Table 4. Incidence and Hazard Ratios of Stroke/SE and MB among Patients with and without prior OAC use for NOACs vs NOACs**

| **No prior OAC use** | **Incidence per 100 person-years** | | **Hazard Ratio**  **95% CI** |  | **Prior OAC use** | **Incidence per 100 person-years** | | **Hazard Ratio**  **95% CI** | **Interaction p-value** |
| --- | --- | --- | --- | --- | --- | --- | --- | --- | --- |
|  |  |  |  |  |  |  |  |  |  |
|  | **Apixaban** | **Dabigatran** |  |  |  | **Apixaban** | **Dabigatran** |  |  |
|  | **(N=3,242)** | **(N=3,261)** |  |  |  | **(N=9,033)** | **(N=9,014)** |  |  |
| Stroke/SE | 1.99 | 2.56 | 0.77 |  | Stroke/SE | 1.76 | 2.61 | 0.68 | 0.5822 |
|  |  |  | (0.52-1.16) |  |  |  |  | (0.54-0.86) |  |
| MB | 8.38 | 7.87 | 1.03 |  | MB | 8.21 | 11.87 | 0.67 | 0.0004 |
|  |  |  | (0.84-1.27) |  |  |  |  | (0.60-0.75) |  |
|  | **Apixaban** | **Rivaroxaban** |  |  |  | **Apixaban** | **Rivaroxaban** |  |  |
|  | **(N=14,426)** | **(N=14,463)** |  |  |  | **(N=20,950)** | **(N=20,913)** |  |  |
| Stroke/SE | 2.36 | 2.66 | 0.89 |  | Stroke/SE | 2.17 | 2.61 | 0.83 | 0.5799 |
|  |  |  | (0.74-1.07) |  |  |  |  | (0.72-0.97) |  |
| MB | 8.43 | 12.99 | 0.64 |  | MB | 9.14 | 13.88 | 0.65 | 0.7445 |
|  |  |  | (0.58-0.70) |  |  |  |  | (0.61-0.70) |  |
|  | **Dabigatran** | **Rivaroxaban** |  |  |  | **Dabigatran** | **Rivaroxaban** |  |  |
|  | **(N=3,253)** | **(N=3,252)** |  |  |  | **(N=9,044)** | **(N=9,045)** |  |  |
| Stroke/SE | 2.56 | 2.62 | 0.99 |  | Stroke/SE | 2.54 | 2.40 | 1.06 | 0.7448 |
|  |  |  | (0.68-1.43) |  |  |  |  | (0.86-1.31) |  |
| MB | 7.84 | 13.06 | 0.62 |  | MB | 11.82 | 13.34 | 0.91 | 0.0003 |
|  |  |  | (0.51-0.75) |  |  |  |  | (0.83-1.00) |  |

**Supplemental Table 5. Incidence and Hazard Ratios of Stroke/SE and MB among Patients with prior GI, ICH, and other bleeds for NOACs vs Warfarin**

| **GI bleed** | **Incidence per 100 person-years** | | **Hazard Ratio** |  | **ICH bleed** | **Incidence per 100 person-years** | | **Hazard Ratio** |  | **Other Bleed** | **Incidence per 100 person-years** | | **Hazard Ratio** | **Interaction**  **p-value** |
| --- | --- | --- | --- | --- | --- | --- | --- | --- | --- | --- | --- | --- | --- | --- |
|  |  |  | **95% CI** |  |  |  |  | **95% CI** |  |  |  |  | **95% CI** |  |
|  | **Apixaban** | **Warfarin** |  |  |  | **Apixaban** | **Warfarin** |  |  |  | **Apixaban** | **Warfarin** |  |  |
|  | **(N=30,668)** | **(N=30,712)** |  |  |  | **(N=7,010)** | **(N=7,017)** |  |  |  | **(N=12,757)** | **(N=12,706)** |  |  |
| Stroke/SE | 2.12 | 3.02 | 0.70 |  | Stroke/SE | 4.64 | 5.46 | 0.85 |  | Stroke/SE | 2.37 | 2.93 | 0.80 | 0.1702 |
|  |  |  | (0.62-0.80) |  |  |  |  | (0.72-1.02) |  |  |  |  | (0.66-0.97) |  |
| MB | 9.93 | 14.40 | 0.68 |  | MB | 5.68 | 8.82 | 0.64 |  | MB | 9.87 | 14.84 | 0.65 | 0.5821 |
|  |  |  | (0.64-0.72) |  |  |  |  | (0.55-0.74) |  |  |  |  | (0.59-0.71) |  |
|  | **Dabigatran** | **Warfarin** |  |  |  | **Dabigatran** | **Warfarin** |  |  |  | **Dabigatran** | **Warfarin** |  |  |
|  | **(N=7,811)** | **(N=7,856)** |  |  |  | **(N=1,592)** | **(N=1,593)** |  |  |  | **(N=3,033)** | **(N=2,987)** |  |  |
| Stroke/SE | 2.44 | 2.53 | 0.97 |  | Stroke/SE | 4.87 | 4.27 | 1.13 |  | Stroke/SE | 1.75 | 2.34 | 0.75 | 0.3417 |
|  |  |  | (0.77-1.21) |  |  |  |  | (0.78-1.65) |  |  |  |  | (0.49-1.13) |  |
| MB | 11.52 | 13.14 | 0.90 |  | MB | 5.94 | 7.81 | 0.76 |  | MB | 11.93 | 14.22 | 0.86 | 0.5867 |
|  |  |  | (0.81-1.00) |  |  |  |  | (0.56-1.04) |  |  |  |  | (0.73-1.02) |  |
|  | **Rivaroxaban** | **Warfarin** |  |  |  | **Rivaroxaban** | **Warfarin** |  |  |  | **Rivaroxaban** | **Warfarin** |  |  |
|  | **(N=22,729)** | **(N=22,785)** |  |  |  | **(N=4,381)** | **(N=4,419)** |  |  |  | **(N=10,295)** | **(N=10,201)** |  |  |
| Stroke/SE | 2.32 | 2.88 | 0.80 |  | Stroke/SE | 4.01 | 5.85 | 0.69 |  | Stroke/SE | 2.15 | 2.52 | 0.85 | 0.3735 |
|  |  |  | (0.70-0.92) |  |  |  |  | (0.55-0.86) |  |  |  |  | (0.69-1.06) |  |
| MB | 14.39 | 13.76 | 1.05 |  | MB | 8.12 | 8.43 | 0.97 |  | MB | 13.63 | 14.39 | 0.94 | 0.1367 |
|  |  |  | (0.99-1.11) |  |  |  |  | (0.82-1.15) |  |  |  |  | (0.86-1.03) |  |

**Supplemental Table 6. Incidence and Hazard Ratios of Stroke/SE and MB among Patients with prior GI, ICH, and other bleeds for NOACs vs NOACs**

| **GI bleed** | **Incidence per 100 person-years** | | **Hazard Ratio** |  | **ICH bleed** | **Incidence per 100 person-years** | | **Hazard Ratio** |  | **Other bleed** | **Incidence per 100 person-years** | | **Hazard Ratio** | **Interaction p-value** |
| --- | --- | --- | --- | --- | --- | --- | --- | --- | --- | --- | --- | --- | --- | --- |
|  |  |  | **95% CI** |  |  |  |  | **95% CI** |  |  |  |  | **95% CI** |  |
|  | **Apixaban** | **Dabigatran** |  |  |  | **Apixaban** | **Dabigatran** |  |  |  | **Apixaban** | **Dabigatran** |  |  |
|  | **(N=7,631)** | **(N=7,723)** |  |  |  | **(N=1,560)** | **(N=1,580)** |  |  |  | **(N=3,084)** | **(N=2,972)** |  |  |
| Stroke/SE | 1.49 | 2.46 | 0.61 |  | Stroke/SE | 3.64 | 4.84 | 0.77 |  | Stroke/SE | 1.64 | 1.80 | 0.90 | 0.3013 |
|  |  |  | (0.46-0.80) |  |  |  |  | (0.51-1.14) |  |  |  |  | (0.57-1.43) |  |
| MB | 9.05 | 11.49 | 0.76 |  | MB | 4.65 | 5.92 | 0.79 |  | MB | 8.27 | 12.00 | 0.66 | 0.3849 |
|  |  |  | (0.68-0.86) |  |  |  |  | (0.55-1.12) |  |  |  |  | (0.54-0.80) |  |
|  | **Apixaban** | **Rivaroxaban** |  |  |  | **Apixaban** | **Rivaroxaban** |  |  |  | **Apixaban** | **Rivaroxaban** |  |  |
|  | **(N=21,584)** | **(N=21,600)** |  |  |  | **(N=4,421)** | **(N=4,352)** |  |  |  | **(N=9,371)** | **(N=9,424)** |  |  |
| Stroke/SE | 1.88 | 2.44 | 0.78 |  | Stroke/SE | 4.26 | 4.13 | 1.03 |  | Stroke/SE | 2.06 | 2.31 | 0.89 | 0.1498 |
|  |  |  | (0.66-0.91) |  |  |  |  | (0.81-1.31) |  |  |  |  | (0.70-1.13) |  |
| MB | 9.63 | 14.54 | 0.65 |  | MB | 5.12 | 8.19 | 0.61 |  | MB | 9.02 | 14.02 | 0.63 | 0.7268 |
|  |  |  | (0.61-0.70) |  |  |  |  | (0.50-0.74) |  |  |  |  | (0.57-0.70) |  |
|  | **Dabigatran** | **Rivaroxaban** |  |  |  | **Dabigatran** | **Rivaroxaban** |  |  |  | **Dabigatran** | **Rivaroxaban** |  |  |
|  | **(N=7,750)** | **(N=7,763)** |  |  |  | **(N=1,548)** | **(N=1,515)** |  |  |  | **(N=2,999)** | **(N=3,019)** |  |  |
| Stroke/SE | 2.42 | 2.24 | 1.08 |  | Stroke/SE | 4.79 | 3.54 | 1.35 |  | Stroke/SE | 1.76 | 2.43 | 0.73 | 0.1052 |
|  |  |  | (0.85-1.38) |  |  |  |  | (0.89-2.03) |  |  |  |  | (0.48-1.10) |  |
| MB | 11.43 | 14.38 | 0.82 |  | MB | 5.99 | 8.65 | 0.69 |  | MB | 11.87 | 12.86 | 0.96 | 0.1291 |
|  |  |  | (0.74-0.91) |  |  |  |  | (0.51-0.94) |  |  |  |  | (0.81-1.14) |  |

**Supplemental Table 7. Hazard Ratios of Stroke/Systemic Embolism and Major Bleeding Among Patients Prescribed Standard Dose NOACs**

|  | **Hazard Ratio (95% CI)** | **p-value** |
| --- | --- | --- |
| **Apixaban vs Warfarin** |  |  |
| Stroke/SE | 0.76 (0.67-0.85) | <0.001 |
| Major Bleeding | 0.69 (0.66-0.74) | <0.001 |
| **Dabigatran vs Warfarin** |  |  |
| Stroke/SE | 1.04 (0.83-1.30) | 0.7382 |
| Major Bleeding | 0.86 (0.77-0.95) | 0.0035 |
| **Rivaroxaban vs Warfarin** |  |  |
| Stroke/SE | 0.73 (0.64-0.84) | <0.001 |
| Major Bleeding | 1.02 (0.96-1.09) | 0.5304 |
| **Apixaban vs Dabigatran** |  |  |
| Stroke/SE | 0.77 (0.66-0.98) | 0.0355 |
| Major Bleeding | 0.78 (0.69-0.88) | <0.001 |
| **Apixaban vs Rivaroxaban** |  |  |
| Stroke/SE | 0.89 (0.76-1.05) | 0.1586 |
| Major Bleeding | 0.68 (0.63-0.73) | <0.001 |
| **Dabigatran vs Rivaroxaban** |  |  |
| Stroke/SE | 1.20 (0.94-1.52) | 0.1378 |
| Major Bleeding | 0.87 (0.78-0.97) | 0.0109 |

**Supplemental Table 8. Hazard Ratios of Stroke/Systemic Embolism and Major Bleeding Among Patients Prescribed Lower Dose NOACs**

|  | **Hazard Ratio (95% CI)** | **p-value** |
| --- | --- | --- |
| **Apixaban vs Warfarin** |  |  |
| Stroke/SE | 0.82 (0.72-0.94) | 0.0046 |
| Major Bleeding | 0.63 (0.59-0.68) | <0.001 |
| **Dabigatran vs Warfarin** |  |  |
| Stroke/SE | 0.91 (0.67-1.22) | 0.5110 |
| Major Bleeding | 0.85 (0.74-0.98) | 0.0272 |
| **Rivaroxaban vs Warfarin** |  |  |
| Stroke/SE | 0.88 (0.75-1.03) | 0.0993 |
| Major Bleeding | 1.09 (1.01-1.17) | 0.0254 |
| **Apixaban vs Dabigatran** |  |  |
| Stroke/SE | 0.73 (0.52-1.03) | 0.0742 |
| Major Bleeding | 0.75 (0.63-0.88) | 0.0005 |
| **Apixaban vs Rivaroxaban** |  |  |
| Stroke/SE | 0.88 (0.74-1.06) | 0.1771 |
| Major Bleeding | 0.59 (0.54-0.64) | <0.001 |
| **Dabigatran vs Rivaroxaban** |  |  |
| Stroke/SE | 1.06 (0.76-1.46) | 0.7406 |
| Major Bleeding | 0.88 (0.76-1.02) | 0.0833 |

**Supplemental Fig 1 Cumulative Incidence of Stroke/Systemic Embolism and Major Bleeding for NOACs vs Warfarin
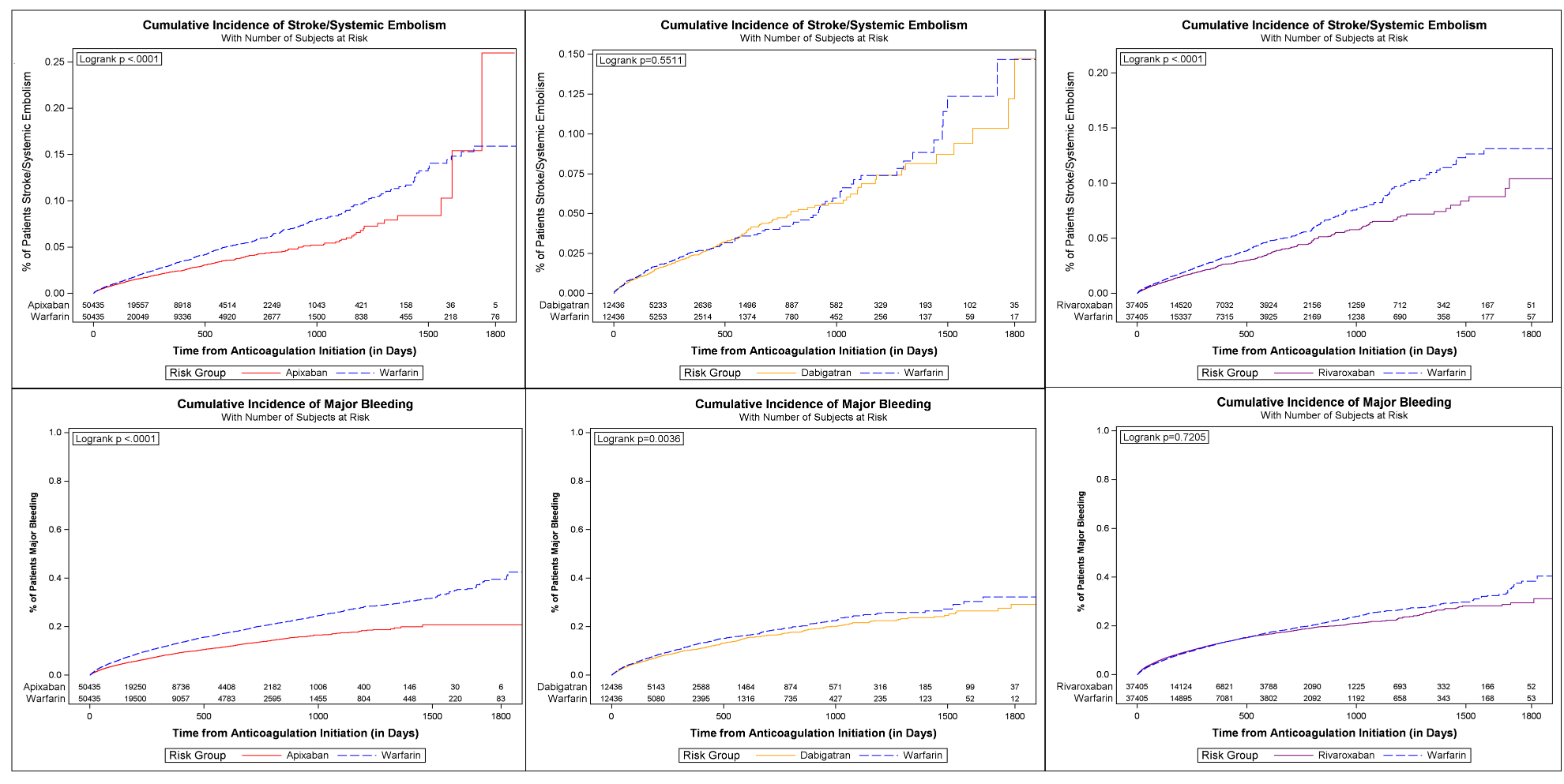
**

**Supplemental Figure 2 Cumulative Incidence of Stroke/Systemic Embolism and Major Bleeding for NOACs vs NOACs**

**
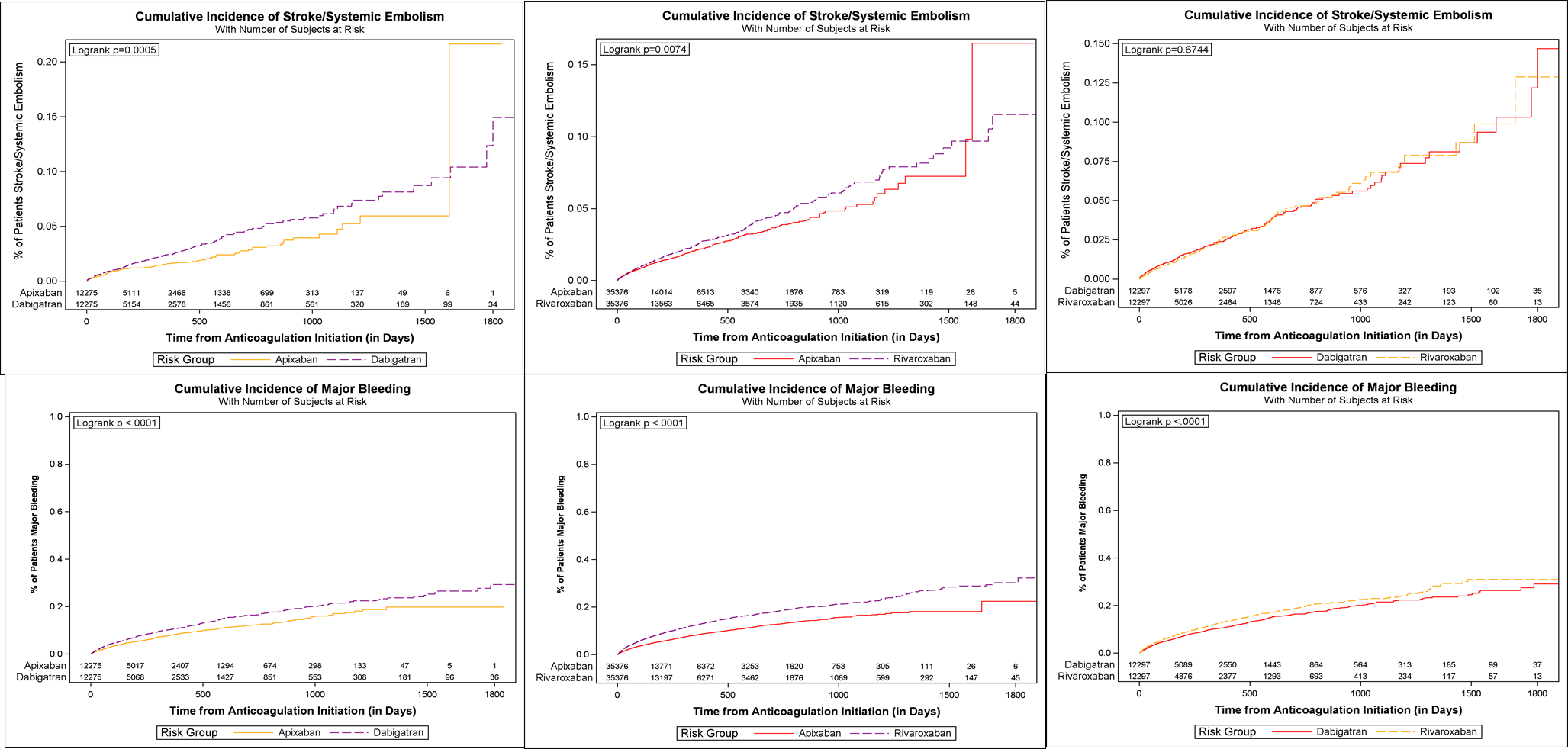
**
